# Supplementary material for: Functional MRI of imprinting memory in awake newborn domestic chicks
Source: Commun Biol. 2024 Oct 15;7:1326. doi: 10.1038/s42003-024-06991-z (PMC11480507; doi:10.1038/s42003-024-06991-z)
Supplement: Supplementary file 2 — Supplementary Information [file 42003_2024_6991_MOESM2_ESM.pdf]

Supporting information for

Functional MRI of imprinting memory in awake newborns domestic chick

Mehdi Behroozi[1,6*], Elena Lorenzi[2,6*], Sepideh Tabrik[3], Martin Tegenthoff[3], Alessandro Gozzi[4], Onur Güntürkün[1,5], Giorgio Vallortigara[2]

Corresponding authors: mehdi.behroozi@ruhr-uni-bochum.de; elena.lorenzi@unitn.it;

Supplementary materials and methods

Supplementary Table 1. Individual data for colour preference after the imprinting experiment.

Subject id	Imprinting colour	First colour presented at test	Distance run toward Red (cm)	Distance run toward Blue (cm)	Ratio preference (approximated)
1	Red	Red	2923	882	0.8
2	Red	Blue	0	0	0.5
3	Red	Red	412	0	1
4	Red	Blue	134	336	0.3
5	Blue	Red	1403	748	0.7
6	Blue	Blue	286	269	0.5
7	Blue	Red	210	34	0.9
8	Blue	Blue	252	470	0.4
9	Red	Red	1714	445	0.8
10	Red	Blue	3553	118	1
11	Red	Red	84	25	0.8
12	Red	Blue	50	0	1
13	Blue	Red	160	0	1
14	Blue	Blue	386	546	0.4
15	Blue	Red	2285	840	0.7
16	Blue	Blue	151	67	0.7
17	Red	Red	17	25	0.4
18	Red	Blue	387	319	0.6
19	Red	Red	1705	302	0.9
20	Red	Blue	588	218	0.7
21	Blue	Red	3902	5813	0.4
22	Blue	Blue	202	2486	0.1
23	Blue	Red	588	395	0.6
24	Blue	Blue	260	378	0.4

Supplementary Table 2. Alphabetised list of abbreviations

Abb.	ROI name	Abb.	ROI name
**Ac**	n. Accumbens	**LH**	Lateral Hypothalamus
**AD**	Dorsal Arcopallium	**LSt**	Lateral Striatum
**AH**	Anterior Hypothalamus	**M**	Mesopallium
**AM**	Medial Arcopallium	**MD**	Mesopallium Dorsale
**APH**	Area parahippocampalis	**MM**	Medial Mesopallium
**Arc**	Arcopallium	**NMm**	nidopallium medial pars medialis
**BNST**	Bed nucleus of the stria terminalis	**MNM**	IMM + MNm
**BOLD**	Blood-Oxygen-Level-Dependent	**MSt**	Medial Striatum
**CDL**	Area Corticoidea dorsolateralis	**N**	Nidopallium
**DLG**	Dorsolateral Geniculate n.	**NCL**	Nidopallium Caudolaterale
**FL**	Field-L	**NCC**	Central Caudal Nidopallium
**fMRI**	functional Magnetic Resonance Imaging	**OV**	n. ovoidalis
**E**	Entopallium	**POA**	Preoptic Area
**GLd**	n. Geniculatus lateralis pars dorsalis	**PoA**	n. posterior pallial amygdala
**HA**	Hyperpallium Apicale	**RARE**	rapid acquisition with relaxation enhancement
**HD**	Hyperpallium Densocellulare	**Rot**	n. Rotundus
**HI**	Hyperpallium Intercalatum	**Rt**	Reticular
**Hp**	Hippocampus	**S**	Septum
**Hp-DM**	Dorsomedial n. of the Hippocampus	**St**	Striatum
**Hp-VM**	Ventromedial n. of Hippocampus	**TeO**	Optic Tectum
**Hyp/H**	Hypothalamus	**Thal**	Thalamus
**IHA**	Interstitial n. of Hyperpallium Apicale	**TnA**	n. Taeniae Amygdalae
**IMM**	Intermediate Medial Mesopallium	**VMH**	Ventromedial hypothalamus
**ITI**	Inter trial interval		

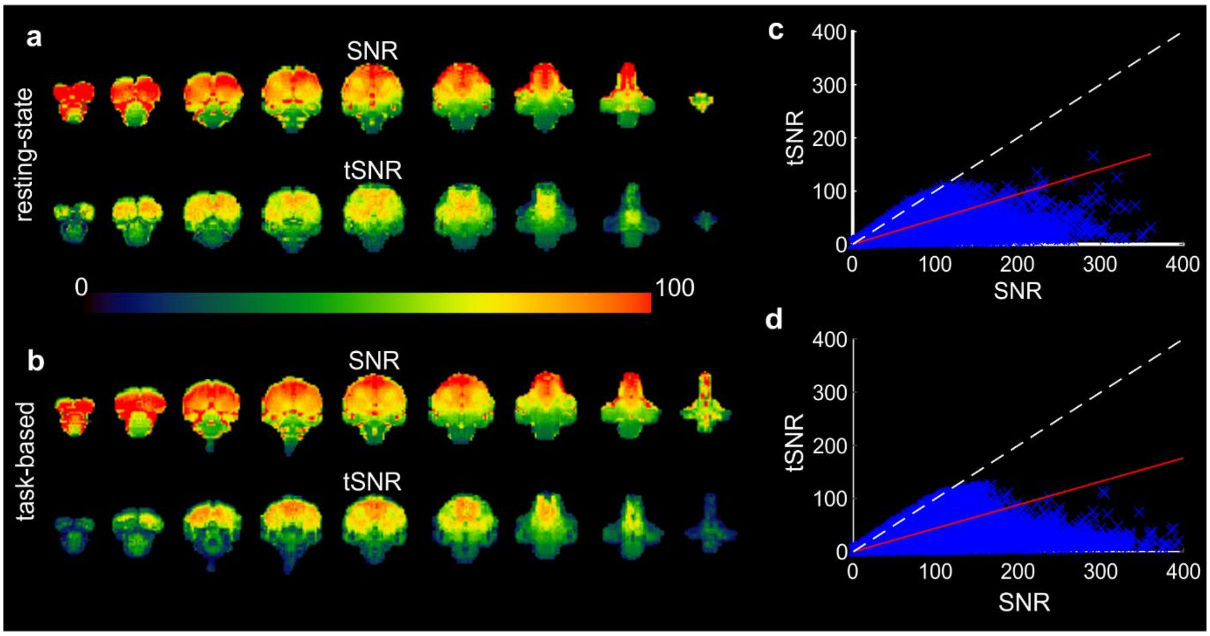

Supplementary Figure 1- *Temporal SNR and SNR assessments. (a,b) tSNR and SNR maps of resting-state and task-based fMRI data. 10 min resting-state data and an imprinting functional scan were used to calculate tSNR. The voxel-wise temporal SNR was defined as the voxel's temporal mean divided by its temporal standard deviation. This figure illustrates that the tSNR (>50) is high in the regions of interest. (c,d) Overall SNR and tSNR were highly correlated with each other during resting-state and task-based fMRI data.*

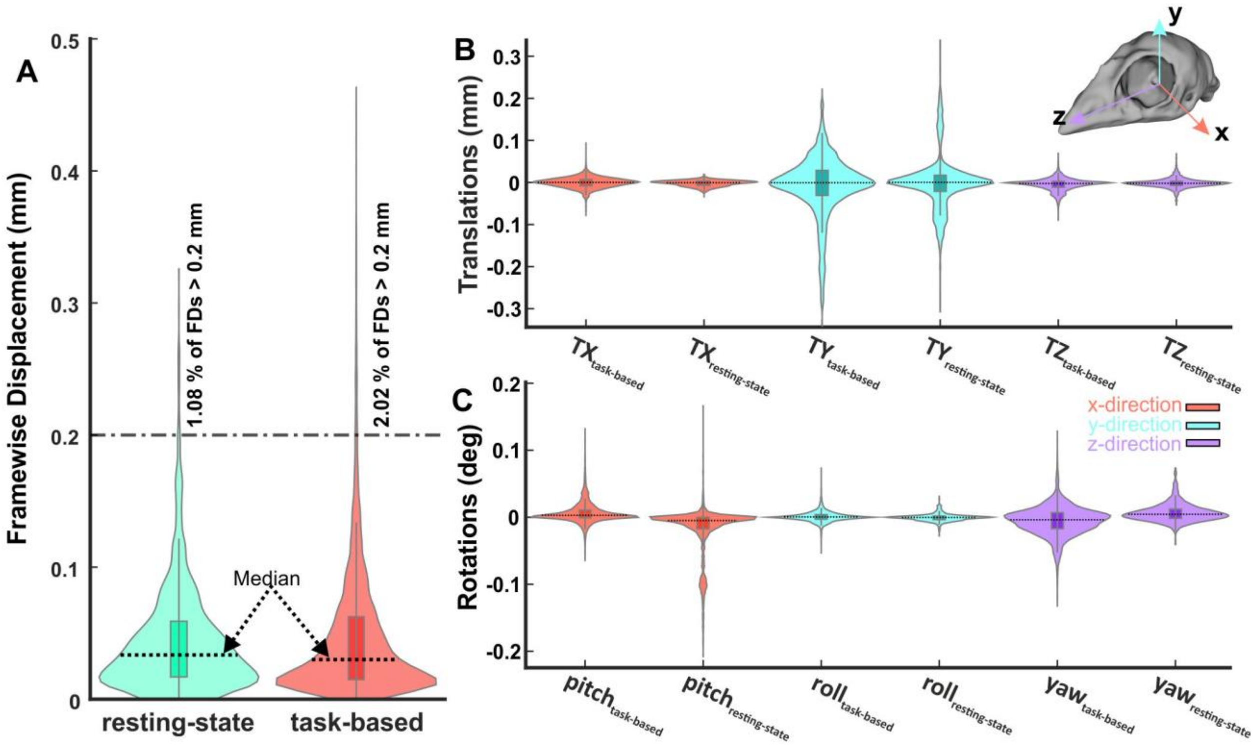

Supplementary Figure 2- *Characterisation of the head movements. (A) Violin plot of the frame-wise displacement over all subjects for task-based (n = 17) and resting-state (n = 9) fMRI scans, respectively. The median is represented by a dash-line across the kernel density estimate of the data. (B, C) Violin plot of estimated motion parameters. Motion parameters were estimated by a 3D rigid body model with six degrees of freedom for translation (x, y, and z-direction) and rotation (pitch, roll, and yaw). The median is represented by a dash-line across the kernel density estimate of the data.*

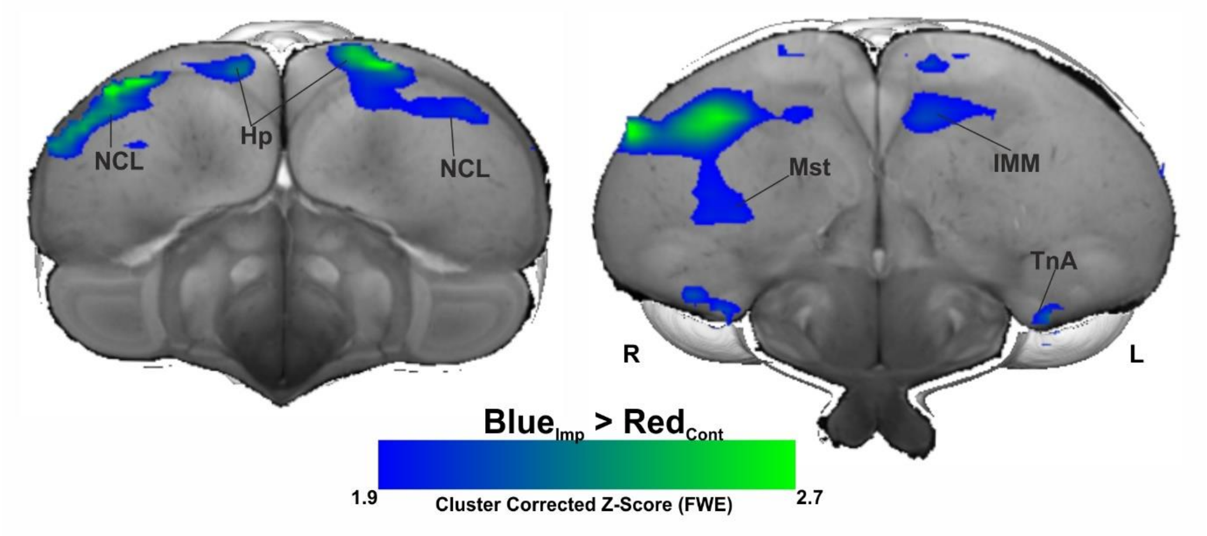

Supplementary Figure 3- *BOLD response pattern during imprinting memory retrieval of blue imprinted colour. The high-resolution coronal slices at the different levels of an ex-vivo chick brain are in greyscale, while the contrast map represents the activation pattern during the presentation of the non-preferred imprinting object after imprinting learning has already occurred (Blue group, the contrast of blue light versus red light conditions during first 10 min of experiment, n = 8, Z = 1.9 and p < 0.05 FEW corrected at the cluster level). The results indicate weak activity in the intermediate medial mesopallium (IMM), the hippocampus (Hp), the medial Striatum (MSt), the nidopallium caudolaterale (NCL) and the nucleus Taeniae of the Amygdala (TnA) when chicks imprinted on blue colour were initially presented to blue. The results are, similar to what happens in red imprinted chicks when presented with red colour (Figure 5). The corresponding abbreviations of ROIs are listed in Table S1*.

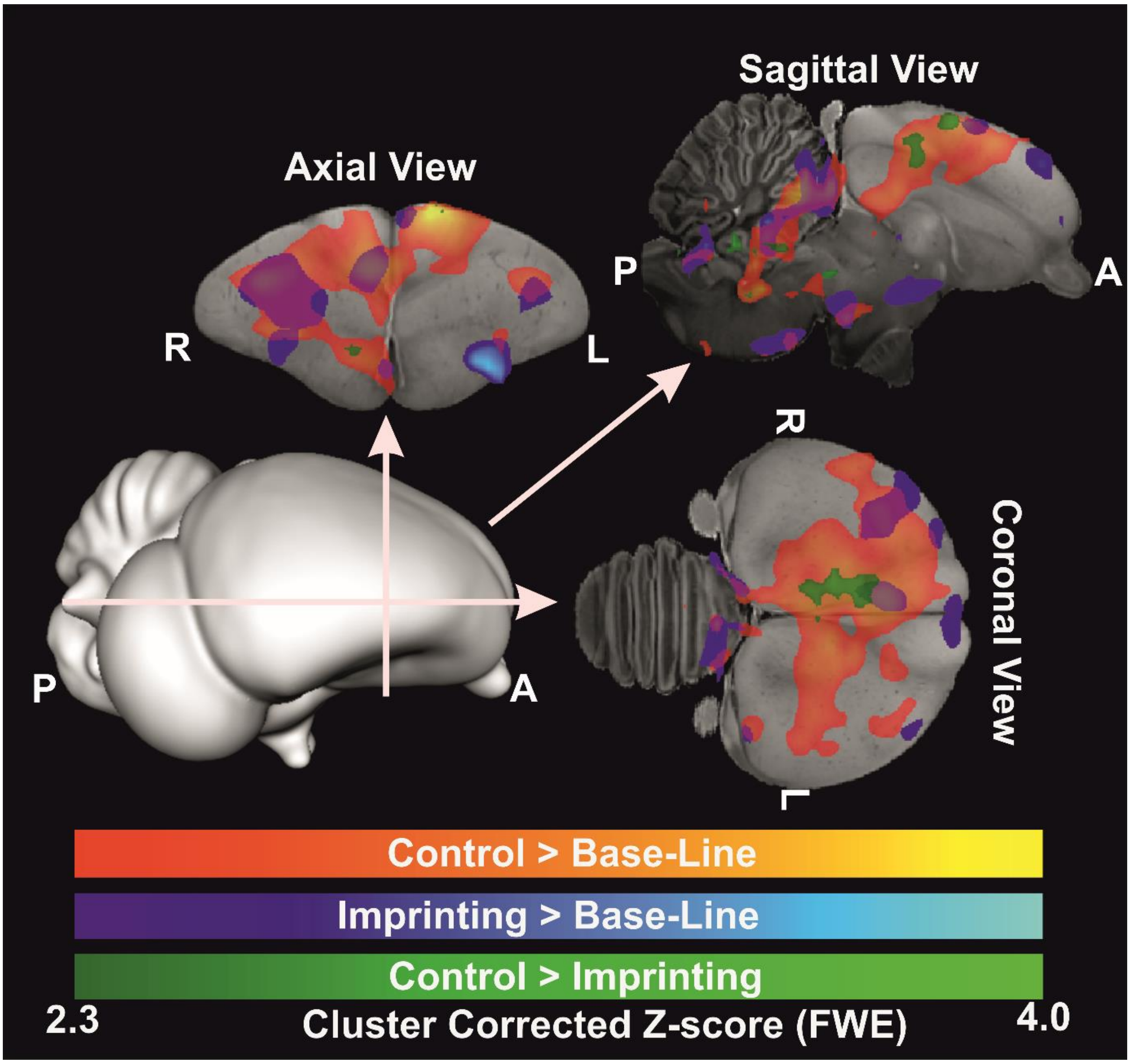

Supplementary Figure 4- *Activation maps for different conditions for Blue Group. Functional activation map from Imprinting and control conditions in the Blue group (last 20 min) show robust activation in the visual pathway and brain areas related to the acquisition of secondary imprinting memory (n = 8, Z = 2.3, and p < 0.05 FEW corrected at the cluster level).*

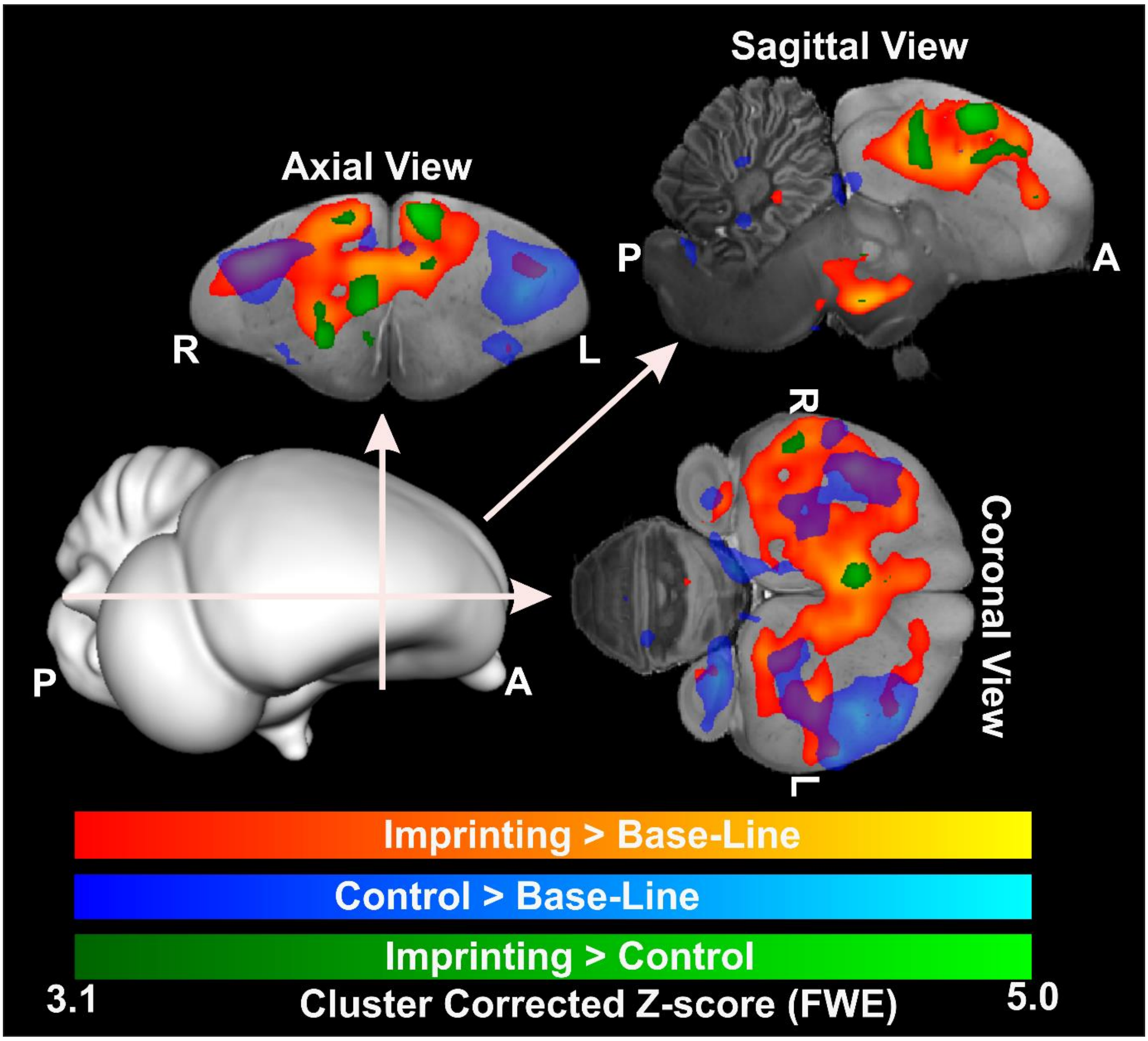

Supplementary Figure 5- *Activation maps for different conditions for Red Group. Functional activation map from Imprinting and control conditions in the Red group show robust activation in the visual pathway and brain areas related to imprinting memory retrieval (n = 9, Z = 3.1 and p < 0.05 FEW corrected at the cluster level).*
