# Supplementary material for: Functional MRI of imprinting memory in awake newborn domestic chicks
Source: Commun Biol. 2024 Oct 15;7:1326. doi: 10.1038/s42003-024-06991-z (PMC11480507; doi:10.1038/s42003-024-06991-z)
Supplement: Supplementary file 3 — Description of Additional Supplementary File [file 42003_2024_6991_MOESM3_ESM.pdf]

# Description Of Additional Supplementary File

**File name:** Supplementary Data 1

**Description:** The source data underlying Supplementary Figure 2.
